# Frailty moderates the relation between moderate-to-vigorous physical activity & stationary time with knee osteoarthritis symptoms

**DOI:** 10.1016/j.tjfa.2025.100077

**Published:** 2025-08-05

**Authors:** Sophie E. Rayner, Selena P. Maxwell, Jocelyn Waghorn, Rebecca Moyer, Kenneth Rockwood, Olga Theou, Myles W. O’Brien

**Affiliations:** a Division of Kinesiology, School of Health and Human Performance, Faculty of Health, Dalhousie University, Halifax, Nova Scotia, Canada; b Geriatric Medicine Research, Dalhousie University & Nova Scotia Health, Halifax, Nova Scotia, Canada; c School of Physiotherapy (Faculty of Health), Dalhousie University, Halifax, Nova Scotia, Canada; d Department of Medicine, Université de Sherbrooke, Sherbrooke, Québec, Canada; e Centre de formation médicale du Nouveau-Brunswick, Université de Sherbrooke & Université de Moncton, Moncton, Canada

**Keywords:** Habitual activity, Accelerometry, Free-living, disease progression, Age-related deficits

## Abstract

Physical activity is protective against osteoarthritic development and is among the best approaches to manage frailty, which can be characterized as the presence of health deficits. It is unclear whether overall health of a person influences the relation between physical activity and knee-joint health.

**Objective:**

Test the hypothesis that physical activity is associated with knee osteoarthritis symptoms and investigate frailty as a moderator.

**Design:**

This cohort observational study included participants (*n* = 1351; 728 females) from the Osteoarthritis Initiative, aged 45–79 (60±9) years. Hip-worn accelerometers were used to quantify free-living stationary time, light (LPA) and moderate-to-vigorous-physical-activity (MVPA). The Western Ontario and McMaster Universities Osteoarthritis Index (WOMAC) defined symptom progression. Baseline frailty was determined via a 31-item Frailty Index and participants grouped into Non-Frail (0–0.09), Very-Mild Frailty (0.1–0.19), and Mild Frailty+ (>0.2). Accelerometry and WOMAC were determined at 72-month follow-up.

**Results:**

MVPA (18±19mins/day) was negatively related to WOMAC outcomes (β<-0.0155, *p* < 0.0022), while LPA (274±79mins/day) was not (β<0.0005, *p* > 0.3061). Stationary time (606±88mins/day) was positively associated with WOMAC stiffness (β=0.0009, *p* = 0.0147). Frailty (0.134±0.077) did not moderate LPA and WOMAC relations (*p* > 0.308). A stronger negative relation between MVPA and WOMAC pain (β=-0.0092, *p* = 0.041) was observed in the Mild Frailty+ group compared to the Very-Mild Frailty and Non-Frail groups. A stronger positive relation between Stationary time and WOMAC stiffness (β=0.0013, *p* = 0.012) was observed in the Mild Frailty+ groups compared to the Very-Mild Frailty and Non-Frail groups.

**Conclusion:**

Engaging in MVPA and limiting stationary time may be more beneficial on knee osteoarthritis pain and stiffness among frailer older adults.

## 1 Introduction

Osteoarthritis is a progressive joint disorder characterized by chronic pain, stiffness, and reduced function that most commonly present in the knee [1]. Aging is among the most impactful risk factors for osteoarthritis with approximately one-fourth of individuals over the age of 40 years having knee osteoarthritis [2], itself a leading cause of disability in older adults [3]. The Osteoarthritis Initiative (OAI), is a longitudinal observational study of middle-aged and older adults created to improve our understanding of osteoarthritis and the factors that influence its development and progression [[4], [5], [6]]. Given the global impact of osteoarthritis on pain and disability [2], investigating modifiable lifestyle factors that may slow/prevent development of symptomatic knee osteoarthritis is needed.

A physically active lifestyle provides numerous health benefits including reducing chronic inflammation [4], and promoting the synthesis of synovial fluid within joints [7], both known to slow the worsening of osteoarthritis [8]. Severe osteoarthritis symptoms create barriers to physical activity, as stiffness and pain during movement may lead to activity avoidance [9]. Physical activity is negatively associated with osteoarthritis symptoms, quantified using the Western Ontario and McMaster Universities Osteoarthritis Index (WOMAC) [10]. The WOMAC consists of three subdomains (pain, stiffness, and physical function/disability); providing an index of osteoarthritis symptom severity [5]. Joint health can be linked to overall well-being, as knee osteoarthritis impacts the individual locally and more broadly, for example through physical activity avoidance which can increase the risk of chronic inflammation [4]. Currently, the impact of the overall health of a person on the strength of physical activity and WOMAC score relation has not yet been investigated.

As osteoarthritis incidence and severity are linked with age [11], it is difficult to investigate the impact of individual conditions that may influence the relation between physical activity and osteoarthritis symptoms, since many health conditions are comorbid with others and are not present in isolation [12]. Frailty can be operationalized as a cumulative decline in physiological reserve that results in the accumulation of health deficits [13] and increases the vulnerability to adverse outcomes (risk of falls, hospital stays, immobilization, disability, etc.) [[14], [15], [16]]. Using a measure of physical frailty [i.e., Fried frailty phenotype [17]], patients with osteoarthritis in the hands, hips, and/or knees were three times more likely to develop frailty compared to non-osteoarthritis patients [18]. Similarly, a frail or pre-frail phenotype in patients with osteoarthritis was associated with worse WOMAC pain scores [18]. Additionally, decreased physical activity is both impactful and characteristic of osteoarthritis and frailty, as persons with osteoarthritis and higher frailty levels engage in less activity [17], [19], and where less physical activity accelerates the development of frailty and osteoarthritis [20]. Frailty is positively associated with inflammation and oxidative stress [12], both predictors of knee pain and osteoarthritis worsening [7]. Conversely, physical activity is known to prevent these age-related processes, indicating that frailty could magnify the positive effects of physical activity on knee osteoarthritis symptoms [21,22]. Potential differences in the relation between different physical activity intensities (light [LPA], moderate-to-vigorous [MVPA]) and both frailty and symptom worsening in osteoarthritis patients has not yet been investigated. The impact of frailty as a multi-dimensional index of organism health on moderating the physical activity and WOMAC relation is unknown but may provide information to support the use of specific activity targets based on the overall health of the individual for the management of knee osteoarthritis symptoms.

We test the hypothesis that physical activity is negatively associated, and stationary time is positively associated, with worse osteoarthritis symptoms. We also hypothesize that frailty moderates these relations, such that higher levels of frailty strengthen the associations between physical activity or stationary time and osteoarthritis severity.

## 2 Methods

### 2.1 Participants

Participants from the OAI were included. The OAI is a multicenter, North American prospective, observational cohort study designed to improve public health through the prevention or alleviation of pain and disability from knee osteoarthritis [23]. The OAI consists of 4796 individuals, with or at risk of knee osteoarthritis. To have been included, participants must have been 45–79 years of age at baseline (September 2006 – July 2008) and have or be at risk for symptomatic knee osteoarthritis. Participants were excluded if they had inflammatory arthritis, contraindication to 3 Tesla Magnetic Resonance Imaging or end-stage bilateral knee osteoarthritis. Detailed inclusion and exclusion criteria have been previously presented [23]. Accelerometer data collections were performed at the 72-month OAI visit to objectively quantify physical activity. Participants were asked to wear the accelerometer during waking hours for one week. Individuals from the OAI who had a 72-month follow-up visit scheduled between September 2012 – July 2014 were included, resulting in 1521 participants who consented to participate in the accelerometry sub-study. Data are publicly available from https://nda.nih.gov/oai. Ethical approval and informed consent were obtained as a part of the original study. The Nova Scotia Health Authority Research Ethics Board does not review research involving secondary analyses of datasets that contain de-identified individual-level data, such as the OAI.

Participants were considered eligible for the current study if they had complete (>80 % of items) frailty data at baseline [24] and wore the accelerometer for at least ≥10 wear hours/day on ≥4 days (*n* *=* 1416/1521). All participants had WOMAC scores at baseline and follow-up. Participants who did not have complete frailty data at baseline (*n* = 41) and those who received knee replacement surgery before or during the 72-month follow up (*n* = 24) were excluded from analysis, resulting in a total of 1351 participants.

### 2.2 Objective physical and stationary activity

Follow-up (72 month) physical activity and stationary activity for each participant was measured using the ActiGraph GT1M uniaxial accelerometer (ActiGraph, Pensacola, USA) worn on their waist to obtain activity counts (sum of accelerations and decelerations along the vertical axis) [25]. The methods for analyzing the accelerometer data output have been previously described in detail [14]. The accelerometers were programmed to collect activity data in 60-second epochs with a sampling frequency of 30 Hz [14]. This study used the National Cancer Institute’s classification of physical activity intensity thresholds with cut points for stationary activity classified as 0–99 activity counts per minute, LPA as 100–2019 activity counts per minute, and MVPA as >2020 activity counts per minute [26]. Since monitors worn on the waist are unable to distinguish quiet standing (not sedentary) from sedentary postures (i.e., sitting/lying), we have opted for the term, “stationary time”. Values for vigorous physical activity were too small to warrant separate analysis, thus moderate and vigorous physical activity were combined (MVPA). No current physical activity intensity cut-offs exist that are specific to osteoarthritis populations, and as such this study has used count-based cut-offs developed from healthy adults, but these likely provide a reasonable measure of higher intensity activity [26].

### 2.3 WOMAC outcomes

All participants completed the WOMAC osteoarthritis index at 72-months follow-up. The WOMAC consists of 24 items within three subdomains: pain (*n* = 5), stiffness (*n* = 2), and physical function or disability (*n* = 17). Questions are scored from 0–4, where: 0 is none, 1 is mild, 2 is moderate, 3 is severe and 4 is extreme symptom severity. Responses are then summed within each subdomain and for total to determine scores, where higher scores indicate more severe symptoms [5]. WOMAC was chosen as the outcome variable as it provides an index of loss of function due to osteoarthritis [5].

### 2.4 Baseline frailty index

The Frailty Index used in the current study was developed specifically for the OAI dataset and validated against mortality records [27]. The Frailty Index was more closely related to mortality than a modified version of the frailty phenotype within the OAI [27]. Standard procedures were followed to calculate baseline frailty [24,28]. The Frailty Index includes 31 items representing different aspects of health, none of which overlapped with the physical activity or WOMAC variables used in the current study (Supplemental Table 1). An absence of the deficit was coded as 0, while presence was coded as 1. Interval or ordinal variables were coded as a fraction of 1, [e.g., None of the time (score: 0.00), Some of the time (0.33), Much of the time (0.67), and All of the time (1.00)]. The Frailty Index for each participant was calculated as the ratio of deficits present to the total deficits assessed, ranging from 0 (no deficits) to 1 (all deficits present). Participants missing >20 % of frailty items (*n* > 6 items missing), were excluded (*n* = 41). Frailty was used to group participants into “Non-Frail” (0–0.09) “Very Mild Frailty” (0.1–0.19) and “Mild Frailty+” (>0.2) subgroups. Mild Frailty (0.2–0.3) and Moderate to Severe Frailty (>0.3) have been separately examined in other studies [27], but due to the limited participants with frailty >0.3 (*n* = 52), the Mild and Moderate to Severe groups were combined as ‘Mild Frailty+’ for the current study. Due to the limited availability of health items measured across timepoints in the OAI, frailty was determined at baseline only.

### 2.5 Statistical analyses

Participant descriptive characteristics were summarized using means and standard deviations (SD) or counts and percentages. Frailty Index (moderator) were extracted at Baseline, while physical activity (independent variable) and WOMAC outcomes (dependent variable) were extracted at 72 months. All analyses were separately conducted for each physical activity variable (LPA, MVPA, and Stationary time) including race (self-reported), sex, and age as covariates.

Multiple linear regressions were performed to test the hypothesis that physical activity was negatively associated with WOMAC scores, and that stationary time was positively associated with WOMAC scores, where higher WOMAC scores are indicative of more severe symptoms. Moderation models were used to test whether the relations between 72-month physical activity (independent variable) and WOMAC scores (dependent variable), were influenced by baseline frailty as a continuous variable. Moderation occurred if the interaction term between the predictor (i.e., physical activity) and moderator (i.e., Frailty) variable was significant. The strength of each relation included in the model was determined via the unstandardized β-values from the regression models [29]. All moderation analyses were adjusted for age, sex, and race, as covariates. Age, sex and race were included as covariates due to existing evidence of associations with knee osteoarthritis [15], [21], but body mass index was not included as a covariate due to multicollinearity concerns as the frailty index included abdominal circumference. Bootstrapped 95 % confidence intervals without 0 for the indirect effect indicates a moderation effect. Simple slopes analyses were performed to investigate any significant interactions, with the independent variable centered to the mean and presented at −1 SD, 0 (mean), and +1 SD, and frailty presented as degree of frailty (non-frail, very mild frailty, and mild+ frailty). All statistical assumptions required to conduct moderation analyses were met. Analyses were completed in SPSS Version 28.0 (IBM, NY, USA), moderation analyses utilized PROCESS script [29]. Descriptive statistics are presented as Mean ± Standard deviation, while regression coefficients are presented as β ± Standard Error (SE). Statistical significance was accepted as *p* < 0.05.

## 3 Results

### 3.1 Participant characteristics

A total of 1351 participants (728 females, Age: 60.6 ± 9.0 years) were included in the analyses. Average baseline frailty was 0.134 ± 0.077, Mean LPA was 274 ± 79 mins/day, MVPA was 18 ± 19 mins/day, and Stationary time was 606 ± 88 mins/day. Participant characteristics by frailty group are presented in Table 1.

**Table 1:** Participant descriptive characteristics by frailty group and in the whole participant group.

	Non-Frail (*n* = 509)	Very Mild Frailty (*n* = 594)	Mild Frailty+ (*n* = 248)	All Participants (*n* = 1351)
Age (years)	57.5 ± 8.4 (45–79)	61.8 ± 8.6 (45–79)*	64.4 ± 9.2 (46–79)*^,^†	60.6 ± 9.0 (45–79)
Females	239 (47 %)	336 (57 %)	153 (62 %)	728 (54 %)
Frailty Index	0.06 ± 0.02 (0–0.09)	0.14 ± 0.03 (0.1–0.19)*	0.26 ± 0.05 (0.20–0.49)*^,^†	0.13 ± 0.08 (0–0.49)
Accelerometer Wear Time (# of days)	6.52 ± 0.73 (4–7)	6.28 ± 0.85 (4–7)*	6.53 ± 0.81 (4–7)†	6.70 ± 0.66 (4–7)
Cohort	432 Incidence (85 %) 4 NE Control (1 %) 73 Progression (14 %)	448 Incidence (75 %) 2 NE Control (<1 %) 144 Progression (24 %)*	154 Incidence (62 %) 0 NE Control (0 %) 94 Progression (38 %)*^,^†	1034 Incidence (77 %) 6 NE Control (<1 %) 311 Progression (23 %)
Race	458 Caucasian (90 %) 51 Non-Caucasian (10 %)	526 Caucasian (89 %) 68 Non-Caucasian (11 %)	182 Caucasian (73 %) 66 Non-Caucasian (27 %)*	1166 Caucasian (86 %) 185 Non-Caucasian (14 %)

Presented as Mean ± SD. NE Control= non-exposed control group.

⁎ Indicates difference vs. Non-Frail (*p* < 0.05).

† indicates difference vs. Very Mild Frailty (*p* < 0.05).

**Fig. 1:**
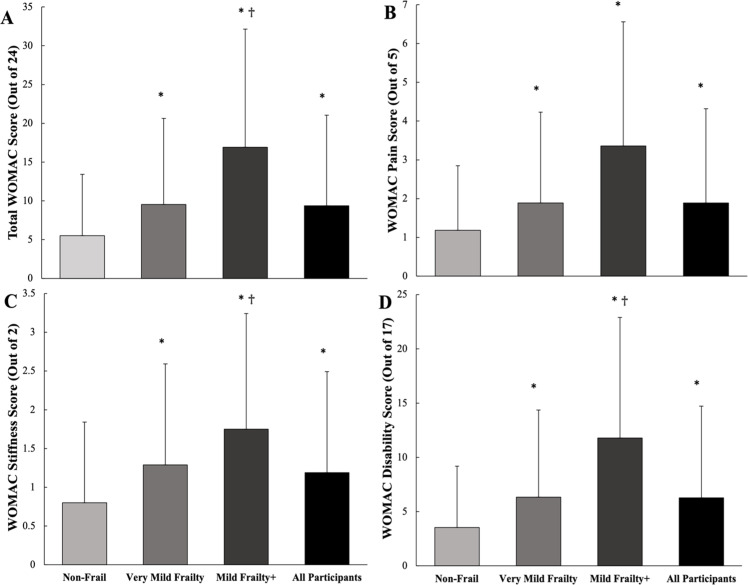
Bar graph depicting 72-month follow-up Western Ontario and McMasters Universities Osteoarthritis Index (WOMAC) scores **A** total, **B** pain, **C** stiffness, and **D** disability, across 3 levels of baseline frailty and the entire sample. * indicates difference (*p* < 0.05) vs. ‘Non-Frail’, † indicates difference (*p* < 0.05) vs. ‘Very Mild Frailty’.

WOMAC scores by frailty group are presented in Fig. 1. Within total WOMAC, Mild Frailty+ group exhibited the highest scores (16.9 ± 15.3), Very Mild Frailty exhibited the middle scores (9.5 ± 11.1) and the Non-Frail group exhibited the lowest scores (5.5 ± 7.9) (all, *p* < 0.001). Similar group differences were also present across the WOMAC pain (Mild Frailty+: 3.4 ± 3.2, Very Mild Frailty: 1.9 ± 2.3, and Non-Frail: 1.2 ± 1.7, *p* < 0.001), stiffness (Mild Frailty+: 1.8 ± 1.5, Very Mild Frailty: 1.3 ± 1.3, and Non-Frail: 0.8 ± 1.0, *p* < 0.001), and disability subdomains (Mild Frailty+: 11.8 ± 11.1, Very Mild Frailty: 6.3 ± 8.0, and Non-Frail: 3.5 ± 5.7, *p* < 0.001)

**Fig. 2:**
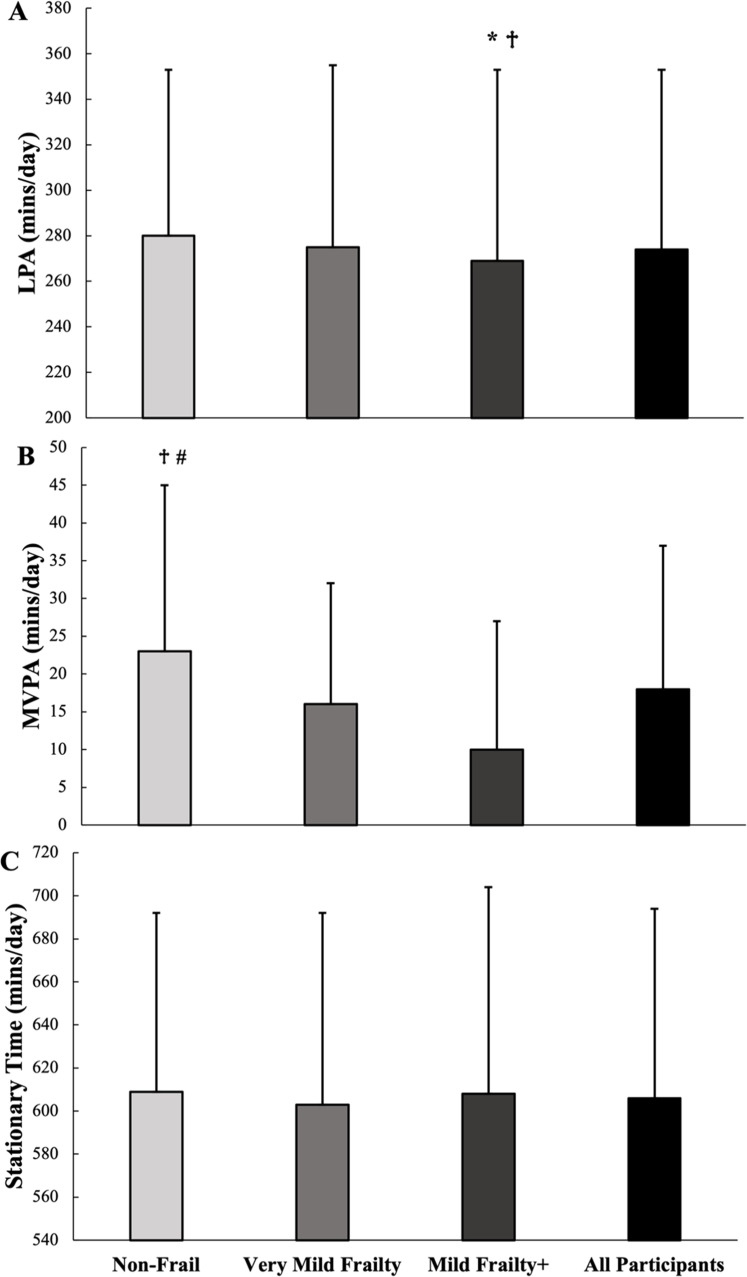
Bar graphs depicting 72 month follow-up physical activity across frailty levels. Accelerometers worn for ≥4 days for ≥10hrs/day. **A:** Average light physical activity (LPA), **B:** Average Moderate-to-Vigorous Physical Activity (MVPA), and **C:** Average Stationary Time. * indicates difference (*p* < 0.05) from ‘Non-Frail’, † indicates difference (*p* < 0.05) from ‘Very Mild Frailty’, # indicates difference (*p* < 0.05) from ‘Mild Frailty+’.

Physical activity outcomes by frailty group are presented in Fig. 2. The ‘Mild Frailty+’ group had less LPA time compared to the ‘Non-Frail’ and ‘Very Mild Frailty’ groups (*p* = 0.047 & *p* = 0.012, respectively), there were no differences between the ‘Non-Frail’ and ‘Very Mild Frailty’ groups (*p* = 1.000). The ‘Non-Frail’ group had more daily MVPA time than the ‘Very Mild Frailty’ and ‘Mild Frailty+’ groups (both, *p* < 0.001), there were no differences between the ‘Very Mild Frailty’ and ‘Mild Frailty+’ groups (*p* = 0.055). There were no differences across all groups for Stationary Time (all, *p* > 0.739).

### 3.2 Associations of frailty, physical activity, and stationary time with WOMAC outcomes

The multiple regressions showed positive main associations of frailty on WOMAC total (total (β=5.3389 ± 0.4368 *p* < 0.001) pain, (β=1.0290± 0.0921, *p* < 0.001), stiffness (β=0.5015 ± 0.0496, *p* < 0.001), and disability (β=3.8068 ± 0.3165, *p* < 0.001). There were no main associations of LPA on WOMAC total (β=−0.0006 ± 0.0039 *p* = 0.875) pain, (β=−0.0002 ± 0.0008, *p* = 0.814), stiffness (β=0.0005 ± 0.0004, *p* = 0.306), or disability (β=−0.0009 ± 0.0028, *p* = 0.742). There were negative main associations of MVPA on WOMAC total (β=−0.0797 ± 0.0173 *p* < 0.001), pain (β=−0.0155 ± 0.0037 *p* < 0.001), stiffness (β=−0.0060 ± 0.0020 *p* = 0.002), and disability (β=−0.0582 ± 0.0125 *p* < 0.001). There was a positive main association of Stationary time on WOMAC stiffness (β=0.0009 ± 0.0004, *p* = 0.015) but not total (β=0.0052 ± 0.0034 *p* = 0.125) pain (β=0.0011 ± 0.0007 *p* = 0.127) or disability (β=0.0032 ± 0.0024 *p* = 0.194).

### 3.3 Frailty as a moderator for physical activity and WOMAC relations

Baseline frailty was investigated as a potential moderator for the LPA, MVPA, and Stationary time relations with WOMAC outcomes. Frailty did not moderate any of the LPA and WOMAC relations (all, β>0.0002, *p* > 0.308).

**Fig. 3:**
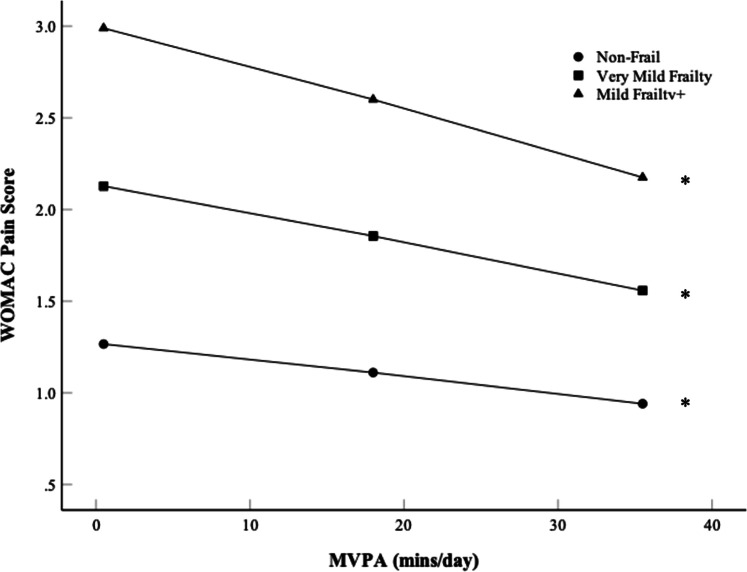
Simple slopes for the moderator variable frailty on the Moderate to Vigorous Physical Activity (MVPA) and Western Ontario and McMaster Universities Osteoarthritis Index (WOMAC) pain score relation. MVPA presented at −1, Mean (18 mins/day), and +1 Standard deviation (SD). Absolute WOMAC pain scores presented (0–5). Frailty grouped by “Non-Frail” (0–0.1), “Very Mild Frailty” (0.1–0.2), and “Mild Frailty+” (>0.2). * Indicates difference in WOMAC pain from −1 SD to +1 SD MVPA (*p* < 0.05).

Frailty moderated the MVPA and WOMAC pain relation (β =−0.0092 ± 0.0045, *p* = 0.041), though frailty was not a moderator across any other WOMAC subdomains. Simple slopes for frailty moderating MVPA and WOMAC pain are presented in Fig. 3. For ‘Non-Frail’, ‘Very Mild Frailty’ and ‘Mild Frailty+’ groups, an increase in daily MVPA decreases WOMAC pain (β =−0.0089 ± 0.0043, *p* = 0.039, β =−0.0155 ± 0.0037, *p* < 0.001, β =−0.0222 ± 0.0054, *p* < 0.001, respectively), where higher frailty increased the strength of the relation (Fig. 3).

**Fig. 4:**
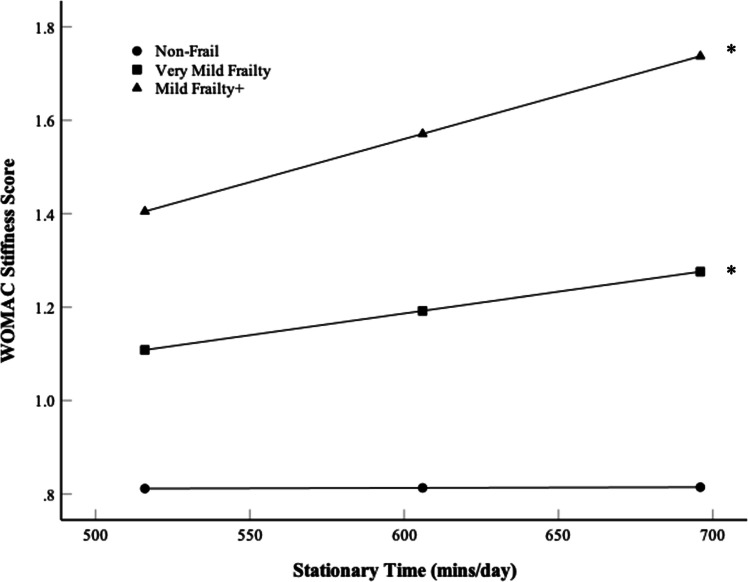
Simple slopes for the moderator variable frailty on the Stationary time and Western Ontario and McMaster Universities Osteoarthritis Index (WOMAC) stiffness score relation. Stationary time presented at −1, Mean (606 mins/day), and +1 Standard deviation (SD). Absolute WOMAC stiffness scores presented (0–2). Frailty grouped by “Non-Frail” (0–0.1), “Very Mild Frailty” (0.1–0.2), and “Mild Frailty+” (>0.2). * Indicates difference in WOMAC stiffness from −1 SD to +1 SD of Stationary time (*p* < 0.05).

Frailty moderated the stationary time and WOMAC stiffness relation (β=0.0013±0.0005, *p* = 0.012) but was not a moderator across WOMAC total (β=0.0102±0.0044, *p* = 0.0933), pain (β=0.0021±0.0009, *p* = 0.125), or disability (β=0.0067±0.0032, *p* = 0.236). For ‘Non-Frail’, ‘Very Mild Frailty’ and ‘Mild Frailty+’ groups, more stationary time increased WOMAC stiffness (β **=**0.0000±0.0005, *p* = 0.9758, β =0.0090 ± 0.0004, *p* = 0.0147, β =0.0018±0.0005, *p* = 0.0004, respectively), where higher frailty increased the strength of the positive relation (Fig. 4).

## 4 Discussion

Here we report a secondary analysis of a large longitudinal observational study of osteoarthritis onset and progression in middle-aged and older adults to test the hypothesis that physical activity (negatively) and stationary time (positively) were associated with two important aspects of osteoarthritis symptom progression (as measured by the WOMAC subdomains scores of pain and stiffness). We found these relations were moderated by frailty: people living with higher degrees of frailty showed greater protection from more physical activity and less stationary time on WOMAC outcomes. Consistent with the hypothesis, frailty was a moderator for the MVPA and WOMAC pain relation, compared to the Very-Mild Frailty and Non-Frail groups there was a stronger negative relation between MVPA and pain among the Mild Frailty+ group, (where higher MVPA time is associated with less severe pain)Additionally, frailty was a moderator for the Stationary time and WOMAC stiffness relation, where there was a stronger positive relation between Stationary time and stiffness among the Mild Frailty+ group. Engaging in MVPA and reducing stationary time may be useful for the prevention and management of knee osteoarthritis related pain and stiffness, particularly among persons who have more health deficits.

Physical activity provides protective effects on osteoarthritis, mitigating several osteoarthritis symptoms [20] and more MVPA time can reduce the risk of developing knee osteoarthritis [30]. The current study observed that frailty moderated the relation between MVPA and WOMAC pain, where MVPA was more beneficial in reducing pain among people with Mild Frailty+ compared to Non-Frail or Very Mild Frailty. This finding supports MVPA as a modifiable, preventative lifestyle factor to slow the progression of osteoarthritis symptoms and is of increased importance for individuals with higher frailty levels. This study proposes that frailty moderates this relation of MVPA but not of LPA due to the greater physiological benefits that the same volume of a higher intensity of exercise provides compared to low-intensity exercise, such as increased lower-body muscle strength, a protective factor for knee osteoarthritis [31]. It is unclear why frailty moderated only the MVPA and WOMAC pain relation but not the stiffness or disability relations, as both MVPA and frailty had main associations on all WOMAC outcomes. Additionally, it must be noted that in our study, an increase in MVPA by 19 mins/day was associated with the MCID in WOMAC pain among individuals with Mild Frailty+ (−0.415) whereas the same difference in WOMAC pain was seen with an increase of 27 and 47 mins/day of MVPA in the Very-Mild-Frailty and Non-Frail groups respectively. These findings suggest that individuals with Mild Frailty+ may experience clinically meaningful improvements in pain with smaller increases in MVPA compared to less frail individuals, indicating that promoting physical activity could yield substantial benefits in vulnerable populations.

It is important to consider the bidirectional relation between physical activity and osteoarthritis symptoms. Although low MVPA may contribute to worsening symptoms, greater pain and stiffness can in turn limit activity levels [10]. Frailty may further moderate this relationship in both directions, as frailer individuals may experience greater functional limitation than non-frail individuals [10]. This suggests that interventions targeting frailty itself may be more effective in enabling increased MVPA and reducing OA symptoms than MVPA-focused interventions alone. The interactions between MVPA and different WOMAC outcomes across frailty groups require further investigation but provide essential information for the development of frailty specific physical activity programs for the prevention/treatment of knee osteoarthritis.

Stationary time is a known risk factor for knee osteoarthritis [32]. Despite increasing risk of knee osteoarthritis, an analysis of the UK Biobank dataset found no differences in objectively measured sedentary time between patients without arthritis and with end-stage arthritis [33]. Similarly, the current study found no differences in Stationary time between the frailty groups. However, frailty did moderate the relation between Stationary time and WOMAC stiffness, where Mild frailty+ increases the harmful effect of Stationary time on WOMAC stiffness compared to Very-Mild Frailty and Non-Frail. We suggest that frailty only moderated the relation between Stationary time and WOMAC stiffness but not the other WOMAC subdomains due to the mechanical secretion process within joint spaces, where movement stimulates synovial fluid production and secretion, lubricating the joint to reduce stiffness [34]. In a stationary joint, this process does not occur, causing drying of the synovial space and accompanied stiffness. It is important to note that an individual can be both physical active (e.g., 30+ mins/day) and accumulate a lot of stationary time (e.g., 600+ mins/day). Also that the frequency of disrupting stationary bouts is more influential to joint health than total movement time [22], where an individual may accumulate MVPA but would not see the reductions in stiffness due to excess stationary time. The processes influencing knee osteoarthritis symptom subdomain progression are complex and require further investigation using interventional models that emphasize reducing and breaking up stationary time.

Although frailty moderated the MVPA and WOMAC pain relation and the Stationary time and WOMAC stiffness relation, there were no interactions with WOMAC total or disability, indicating that frailty plays a role in the physical activity and osteoarthritis symptom progression relation but is not the only factor modulating these relations. Higher MVPA was associated with lower WOMAC outcomes in all groups, while less stationary time was associated only with lower stiffness scores across all groups. Thus, increasing MVPA and decreasing stationary time in individuals with frailty and with/at high risk of knee osteoarthritis may aid in slowing the worsening of symptom severity, but not completely prevent symptom progression. We suggest that instead of increasing MVPA and reducing stationary time independently of one another, replacing stationary time with MVPA may provide a synergistic effect on knee osteoarthritis symptoms, especially among persons who have accumulated health deficits. Previous evidence suggests that MVPA is both safe and beneficial for frail individuals when it is tailored to the population and appropriately supervised [35]. Strategies to help this more vulnerable population achieve this are needed. In age-related diseases, such as osteoarthritis, there are a multitude of factors that influence the progression of both frailty and the disease itself. For example, structural bone defects lead to increased risk of knee osteoarthritis severity, but are not impacted by frailty or physical activity [36]. Nevertheless, frailty provides a comprehensive, holistic index of overall health, that may provide useful aging-related information beyond chronological years alone [37]. To create effective protocols to reduce the incidence and slow progression of knee osteoarthritis related-disability, there is not one sole factor of importance, but rather a multitude [12]. Identifying frailty as a moderator for the MVPA/Stationary time and WOMAC relations provides evidence to include higher-intensity habitual activity and sedentary behaviour interventions in treatment/prevention protocols for frailer adults.

Adding to the literature, this study investigated frailty as a moderator for the objective 72-month physical activity and WOMAC outcome relations in the OAI. Though, the study is not without its limitations. The participants studied consisted of predominantly white individuals (86 %), limiting usefulness for more diverse populations. The analytic sample was drawn from all cohorts (incidence, progression, and non-exposed controls) of the OAI. This approach allowed us to examine not only symptomatic changes among those with established OA but also early symptom development in individuals prior to disease development. Additionally, there were few individuals of moderate to high frailty (*n* = 52). Frailty data were self-reported and only available at baseline and not at the 72-month follow-up, preventing analyses on trajectories of frailty over time, but baseline frailty levels are the best predictor of future frailty level [27]. Further, the hip-worn accelerometers cannot provide true sedentary time measures since they cannot distinguish the orientation of the thigh but can provide insight into stationary time. Moreover, detailed accelerometer-derived outcomes, such as activity counts, mean absolute deviation, and Euclidean Norm Minus One (ENMO) were not available, limiting the precision of physical activity characterization. Despite these limitations, the study had a large sample of 1351 individuals with baseline frailty, 72-month follow-up habitual activity data, and WOMAC scores necessary to answer our proposed research question.

In conclusion, the protective effect of increased physical activity for WOMAC pain and reduced stationary time on WOMAC stiffness was greatest among people with Mild Frailty+ (frailty: >0.20) compared to people living with Very Mild Frailty or those who were Non-Frail. These findings provide support for MVPA-focused exercise prescription and sedentary behaviour interventions for individuals with Mild Frailty+ and with/at high risk of knee osteoarthritis to pain and stiffness progression.

## Funding

SPM was supported by a CIHR Post-Doctoral Fellowship Award (#494,621) and a Dalhousie University Department of Medicine University Internal Medicine Research Foundation Research Fellowship Award.

## Conflicts of interest

KR has asserted copyright of the Clinical Frailty Scale and KR and OT have asserted copyright of the Pictorial Fit-Frail Scale which are both made freely available for education, research, and not-for-profit health care. Licenses for commercial use of the Clinical Frailty Scale and the Pictorial Fit-Frail Scale are facilitated through the Dalhousie Office of Commercialization and Industry Engagement. Additionally, KR is the Co-founder of Ardea Outcomes (until 2021 DGI Clinical) which has held contracts with Novartis, among other pharma and device manufacturers, within the last three years. All other authors have no conflicts of interest to report.

## CRediT authorship contribution statement

**Sophie E. Rayner:** Writing – review & editing, Writing – original draft, Visualization, Methodology, Investigation, Formal analysis, Data curation, Conceptualization. **Selena P. Maxwell:** Investigation, Formal analysis. **Jocelyn Waghorn:** Writing – review & editing. **Rebecca Moyer:** Writing – review & editing. **Kenneth Rockwood:** Writing – review & editing. **Olga Theou:** Writing – review & editing. **Myles W. O’Brien:** Writing – review & editing, Validation, Formal analysis, Conceptualization.

## Declaration of competing interest

The authors declare the following financial interests/personal relationships which may be considered as potential competing interests: Selena P. Maxwell reports a relationship with 10.13039/501100000024Canadian Institutes of Health Research that includes: funding grants. Selena P. Maxwell reports a relationship with 10.13039/501100005785Dalhousie University Faculty of Medicine that includes: funding grants. Kenneth Rockwood has patent Clinical Frailty Scale licensed to Geriatric Medicine Research Team. Kenneth Rockwood and Olga Theou has patent Pictoral Fit-Frail Scale licensed to Geriatric Medicine Research Team. Kenneth Rockwood is the Co-founder of Ardea Outcomes (until 2021 DGI Clinical) which has held contracts with Novartis, among other pharma and device manufacturers, within the last three years. If there are other authors, they declare that they have no known competing financial interests or personal relationships that could have appeared to influence the work reported in this paper.

## Appendix Supplementary materials

Image, application 1
