# Supplementary material for: Frailty moderates the relation between moderate-to-vigorous physical activity & stationary time with knee osteoarthritis symptoms
Source: J Frailty Aging. 2025 Aug 5;14(5):100077. doi: 10.1016/j.tjfa.2025.100077 (PMC12702437; doi:10.1016/j.tjfa.2025.100077)
Supplement: Supplementary file 1 [file mmc1.docx]

SUPPLEMENTAL TABLES

**Supplemental Table 1** Items included in the frailty index.

| *Comorbidities:* | *Function:* |
| --- | --- |
| - Cancer history | - Health limits activities |
| - Diabetes | - Health limits stairs |
| - Heart attack | - Health results in accomplishing less |
| - Heart failure | - Health limits kind of work |
| - Stroke - Blocked artery in leg | - 400 m walk - 5x chair stand |
| - Kidney disease |  |
| - Lung disease |  |
| - Ulcers | *Vital Signs:* |
| - Gout | - Systolic blood pressure |
| - Other arthritis* | - Low diastolic blood pressure |
| - Broken/fractured bones after 45 y | - Pulse pressure |
| - Broken/fractured spine | - Radial pulse |
| - Abdominal circumference |  |
|  |  |
|  | *Other:* |
| *Mental health:* | - Polypharmacy |
| - Everything feels like an effort | - Self-reported health |
| - Loneliness - Could not “get going” - Feels as good as others |  |
| - Hopeful for the future |  |
|  |  |

*Arthritis types other than osteoarthritis and rheumatoid arthritis
